# Effectiveness of high-sensitivity troponin I to predict cardiovascular events in chest trauma patients

**DOI:** 10.1007/s00068-025-03065-5

**Published:** 2026-01-29

**Authors:** Baptiste Milley, Xavier-Jean Taverna, Stanislas Abrard, Sophie Debord-Peguet, François-Xavier Jean, Régine Cartier, Anne-Claire Lukaszewicz, Pauline Pérèz

**Affiliations:** 1 Anaesthesia and Critical Care Medicine Department, Hospices Civils de Lyon, Edouard Herriot Hospital, Lyon, France; 2 Centre de Biologie Est, Groupement Hospitalier Est, Hospices Civils de Lyon, Bron, France; 3 EA 7426, Pathophysiology of Injury-Induced Immunosuppression, (Université Claude Bernard Lyon 1 -Hospices Civils de Lyon - bioMérieux), Edouard Herriot Hospital, Lyon, France

**Keywords:** Chest trauma, Blunt cardiac injury, High-sensitivity troponin, Cardiac biomarkers, Risk stratification, Intensive care

## Abstract

**Background:**

Chest trauma accounts for nearly one-third of severe trauma cases and is associated with cardiac complications that increase morbidity and mortality. The utility of repeated high-sensitivity troponin I (hsTnI) testing in this context remains uncertain, particularly with the advent of ultra-sensitive assays.

**Methods:**

We conducted a retrospective cohort study of 1,749 intensive care patients with chest trauma who underwent hsTnI testing. Cardiac complications during intensive care hospitalisation and 30-day mortality were recorded. Predictive performance of initial hsTnI was assessed using receiver operating characteristic curve (ROC) analysis, and associations between hsTnI values (initial and dynamic changes) and complications were tested in a multivariate logistic regression model adjusted for age and severity (SAPS II Score).

**Results:**

Cardiac complications occurred in 64 patients (3.7%), most frequently arrhythmias (1.6%). Initial hsTnI showed moderate predictive value (AUROC 0.74), with a threshold of 12.9 ng/L providing 73% sensitivity and 71% specificity. Patients with complications were older, had higher SAPS II scores, prolonged ICU stays, greater need for organ support, and increased 30-day mortality. In a multivariate model, neither initial hsTnI nor its variation independently predicted complications, whereas age and SAPS II remained significant predictors.

**Conclusion:**

Cardiac complications after thoracic trauma are infrequent but strongly associated with adverse outcomes. Initial hsTnI offers only moderate prognostic performance, and a second testing does not improve risk stratification. Age and severity scores are stronger predictors of complications in this setting.

## Introduction

Blunt chest trauma is a common issue in trauma patients, affecting up to one third of them [1], and is associated with significant morbidity and mortality [2, 3]. Among these patients, blunt cardiac injury (BCI) is a major concern for physicians due to its potential association with serious haemodynamic and rhythm complications. Reported BCI prevalence ranges from 3 to 56% [4], mainly because of the wide range of definitions in the literature. These definitions encompass diverse clinical or biological findings, with debated clinical relevance, such as asymptomatic electrocardiographic changes. No diagnostic gold standard exists for BCI; clinicians therefore use a composite of bedside assessment, electrocardiography (ECG) and measurement of cardiac biomarkers, mainly cardiac troponin I (cTnI) - as endorsed by the Eastern Association for the Surgery of Trauma (EAST) guidelines [5] - or troponin T (cTnT). Cardiac troponin I (cTnI) and T (cTnT) are proteins involved normally in contraction of cardiomyocytes and are thus specific of cardiac tissue lesions. Consequently, many studies have incorporated cTnI measurement in BCI diagnosis. When the ECG is normal, an undetectable cTnI confers a 100% sensitivity for excluding BCI; however, its capacity to predict subsequent cardiac events is more controversial [5]. High-sensitivity troponin (hsTn) assays have supplanted conventional methods and are already widely used in the diagnosis of myocardial infarction (MI) where a decrease in hsTn levels over a short period of time usually rule out MI [6, 7]. These ultra-sensitive techniques enable lower thresholds and finer levels of variation to be detected, and this more sensitive detection may thus result in a higher proportion of asymptomatic positive patients [8]. In the trauma setting, one study suggested that high sensitivity troponin I (hsTnI) was comparable with troponin I (TnI) for the diagnosis of clinically significant BCI [9]. However, many of these studies are limited by the small number of patients included and the variability of definitions for clinically significant BCI, including minor events such as isolated ECG alterations, mild biomarker rises or non-specific symptoms (e.g., chest pain, transient hypotension). Moreover, in the few studies which evaluate use of ultrasensitive assays, none focus on the dynamic changes of hsTnI value over time despite more precise detection of blood levels. In this context, our study aimed to evaluate the predictive value of hsTnI initial value and its variation on major cardiac events in a large cohort of chest trauma patients admitted to ICU. We hypothesised that hsTnI levels and their variation would be poor predictors of clinically relevant cardiac events in the population of chest trauma. We hypothesised that neither baseline hsTnI level nor its variation would reliably predict clinically significant cardiac events after chest trauma.

## Methods

### Study participants

We performed a retrospective observational study using patients records from a Level 1 academic trauma-centre intensive care unit (ICU). All consecutive ICU admissions for trauma between December 2017 and December 2022 with an ICD-10 diagnosis related to chest injury were screened for inclusion. Patients were included if at least one high-sensitivity troponin I (hsTnI) measurement had been performed. The study was approved by the local ethics committee (Hospices Civils de Lyon), and informed consent was waived due to the retrospective nature of the research.

#### Data collection and procedures

Demographic and clinical data were collected retrospectively from electronic health records, including age, sex, type of thoracic lesions, ICU length of stay (LOS), mechanical ventilation duration, vasopressor use, and renal replacement therapy. ICD-10 diagnostic codes and SAPS II scores (Simplified Acute Physiology Score II) were extracted from the French hospital discharge database (PMSI). Laboratory data, including hsTnI measurements, were retrieved from the hospital laboratory system. The assay method for hsTnI remained unchanged throughout the study period, ensuring consistency in measurements. The first hsTnI assay was performed upon admission of patients with chest trauma to the resuscitation room or the emergency department. Otherwise, when it was not performed at the admission, it was included in the first ICU biological assessment. On admission to the ICU, ECG and chest point-of-care ultrasound (POCUS) were performed routinely, according to the local protocol. However, examinations performed during the remaining ICU stay were at the discretion of clinicians, based on previous results or clinical presentation.

Electronic medical records were reviewed to identify hospital admissions using ICD-10 diagnostic codes associated with cardiac events. For each patient identified, their hospitalisation reports were examined to determine the specific type of cardiac event. Clinically significant cardiac event was defined as one of the following diagnostic criteria: 1 - Abnormal ECG rhythm or conduction, including new-onset atrial fibrillation, bundle branch or atrioventricular block, wide QRS tachycardia or an acute ST segment or T wave change; 2 - Echocardiographic abnormalities including pericardial effusions, ventricular wall motion abnormalities, left or right ventricular failure, or acute valvular dysfunction; 3 - Cardiac arrest reported as unexpected, excluding cardiac arrest in a context of withholding or withdrawal of supportive care. Mortality status was checked through the national demographic registry maintained by the French National Institute of Statistics and Economic Studies (INSEE).

#### Statistical analyses

Data were stored in Microsoft Excel. Statistical analyses and figures were generated with R (version 4.4.0, R Foundation for Statistical Computing, Vienna, Austria). The distribution of the continuous variables was assessed by histograms. Continuous data are expressed as median and interquartile range (IQR). Comparisons between groups were assessed using Student t-test tests for continuous variables and Chi-squared or Fisher’s exact test for categorical variables when appropriate. Multivariate analysis was performed using a logistic regression model to identify factors independently associated with the outcomes. Receiver operating characteristic (ROC) curves assessed the ability of hsTnI to discriminate significant cardiac events and 30-day mortality. The Youden index and area under the curve (AUC) were computed for each ROC analysis.

**Fig. 1:**
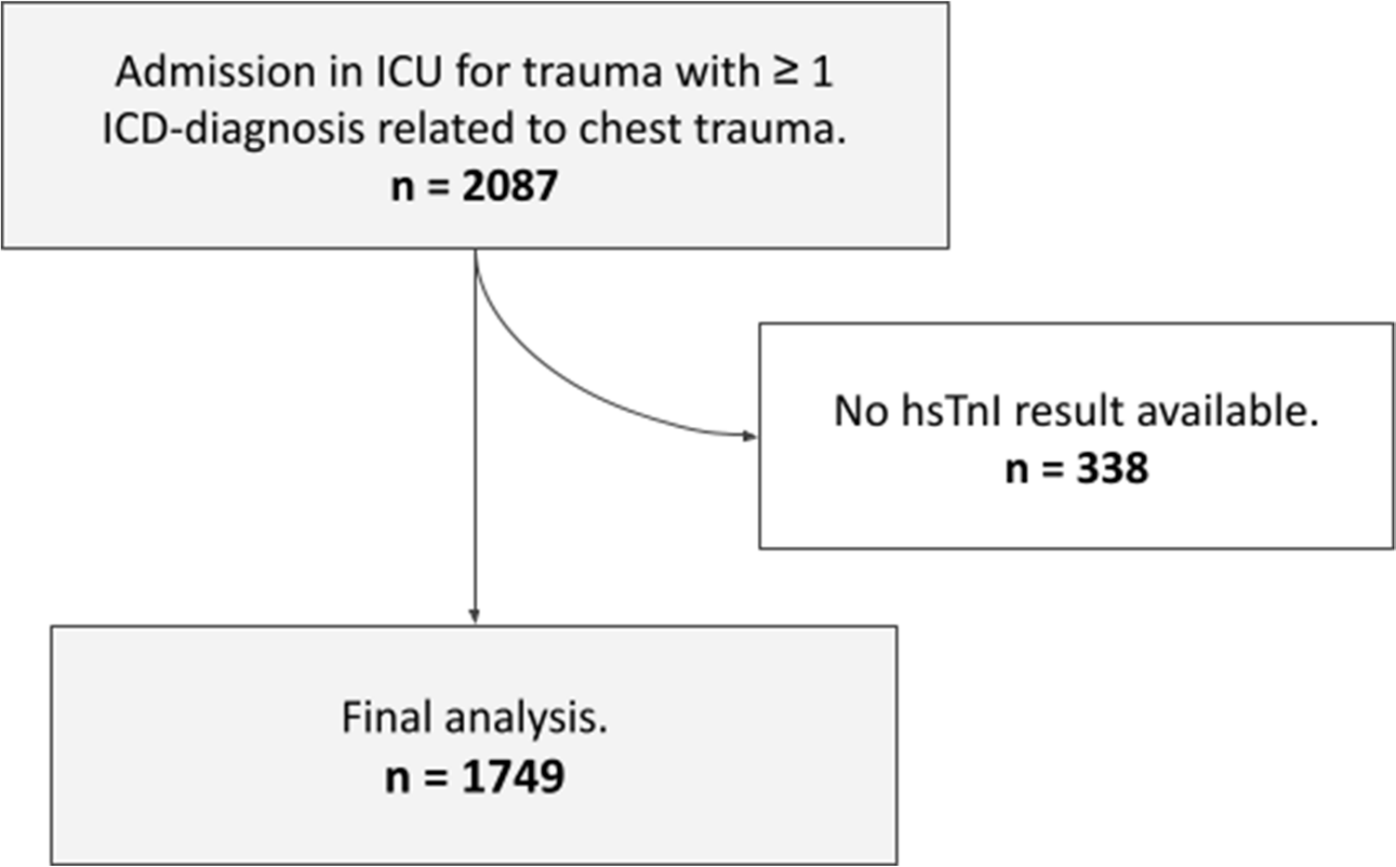
Study flow diagram of ICU admissions for thoracic trauma from December 2017 to December 2022 at a Level 1 trauma centre ICU

## Results

During the five-year study period, 2,087 patients were admitted to the ICU with ICD-10 diagnostic codes for thoracic trauma; 338 were excluded due to unavailable hsTnI measurements (Fig. 1). Ultimately, 1,749 patients were included in the final analysis. Among these patients, the first hsTnI assay was performed a median of 1.08 hours before ICU admission (IQR 1.58 - 0.42 hours before admission). Patient demographics and baseline characteristics are summarised in Table 1. Of the 1,749 patients included, 64 experienced major cardiac events, yielding an incidence of 3.7% (64/1,749). The most frequent event was arrhythmia (1.6%, 28/1,749), followed by unexpected cardiac arrest (0.7%, 13/1,749), delayed pericardial effusion (0.7%, 12/1,749), and acute heart failure (0.6%, 11/1,749). Only five patients developed acute valvular dysfunction (0.3%, 5/1,749). Thirty-day all-cause mortality was 6.3% (111/1,749). Event rates are detailed in Table 2.

**Table 1:** Baseline characteristics of the ICU chest trauma cohort overall and stratified by the occurrence of major cardiac events. Data are presented as median (interquartile range) or n (%)

Characteristic	Overall	No cardiac event	Cardiac event
	N = 1,749^*1*^	N = 1,684^*1*^	N = 64^*1*^
Age (years)	42 (27, 59)	42 (26, 58)	65 (49, 81)
Sex (male)	1,328 (76%)	1,281 (76%)	47 (73%)
Organ support therapies			
Vasopressor drug use	545 (31%)	506 (30%)	39 (61%)
Renal replacement therapy	70 (4.0%)	60 (3.6%)	10 (16%)
Invasive ventilation	466 (27%)	429 (25%)	37 (58%)
Non-invasive ventilation	805 (46%)	768 (46%)	37 (58%)
Thoracic lesions			
1 rib fracture	150 (8.6%)	148 (8.8%)	2 (3.1%)
$\ge$ 2 rib fractures	737 (42%)	703 (42%)	34 (53%)
Pneumothorax	531 (30%)	510 (30%)	21 (33%)
Hemothorax	386 (22%)	368 (22%)	18 (28%)
Flail chest	152 (8.7%)	145 (8.6%)	7 (11%)
Sternal fracture	115 (6.6%)	108 (6.4%)	7 (11%)
SAPS II Score	24 (15, 39)	23 (15, 38)	48 (32, 67)
Initial hsTnI (ng/L)	5 (5, 21)	5 (5, 19)	34 (10, 166)

^*1*^Median (Q1, Q3); n (%)

**Table 2:** Incidence of major cardiac events during hospital admission and 30‑day all-cause mortality in the study cohort. Events comprise arrhythmia, myocardial ischaemia, pericardial effusion, acute valvular dysfunction, acute heart failure, and unexpected cardiac arrest, reported as counts and percentages

Characteristic	N = 1,749^*1*^
Arrhythmia	28 (1.6%)
Myocardial ischaemia	3 (0.2%)
Pericardial effusion	12 (0.7%)
Acute valvular dysfunction	5 (0.3%)
Acute heart failure	11 (0.6%)
Cardiac arrest	13 (0.7%)
30-day mortality	111 (6.3%)

^*1*^n (%)

### Predictive value of high sensitivity troponin I

**Fig. 2:**
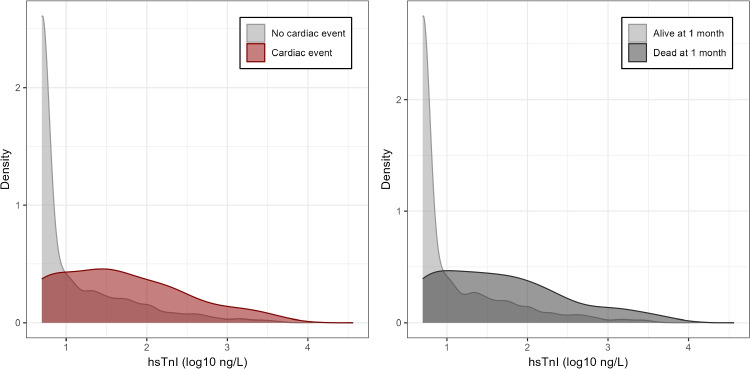
Distribution of initial hsTnI levels according to occurrence of cardiac event and 1-month mortality in the chest trauma cohort. Each panel represent density plots, allowing a smoothed estimation of the underlying distribution of log-transformed hsTnI levels (log10 ng/L) within each patient group. In the left panel, patients are stratified according to the occurrence or not of a cardiac event during ICU stay. In the right panel, patients are stratified according to their vital status, alive or dead, 1 month after ICU admission

**Fig. 3:**
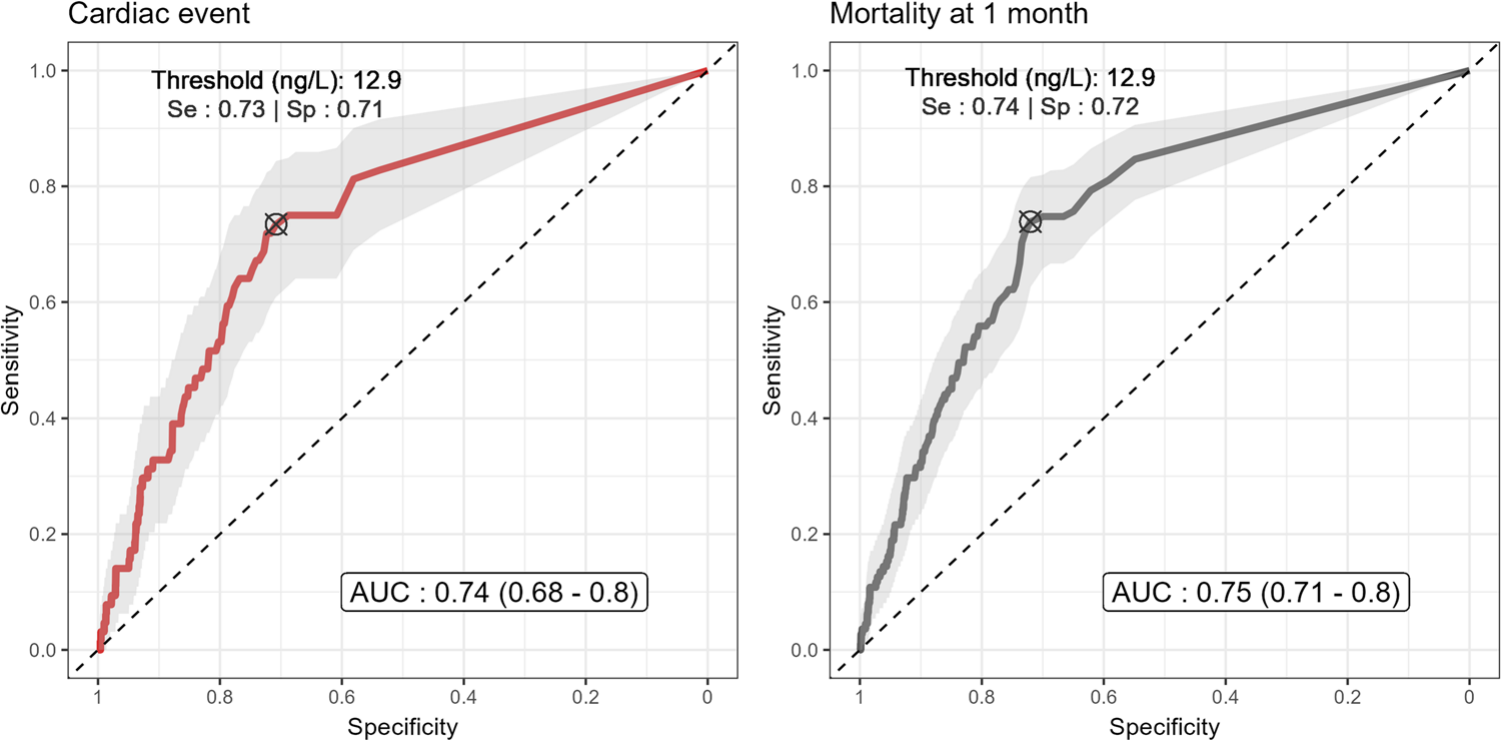
Receiver-operating-characteristic (ROC) curves for initial hsTnI level to discriminate cardiac event during ICU stay and 30‑day mortality in the chest trauma cohort. The plot displays the specificity (x-axis) and the sensitivity (y-axis) of hsTnI to predict either a cardiac event (left panel) or mortality at 1 month (right panel) across all possible decision thresholds. The cross-in-circle marker denotes the threshold value with the highest Youden index, maximising sensitivity and specificity. The area under the ROC curve (AUC) quantifies the overall ability of the model to discriminate between patients with and without the outcome, with higher values indicating better discrimination

The laboratory results were used to assess predictive value of hsTnI on prediction of significant cardiac event during admission or mortality at 1 month. Using this data, hsTnI concentrations were evaluated continuously to generate histograms and receiver operating characteristic (ROC) curves for each outcome. The distribution of hsTnI concentrations according to patient outcomes (major cardiac events and 30-day mortality) is presented in Fig. 2. ROC curves for TnI discrimination of cardiac events and 30-day mortality are displayed in Fig. 3. Initial hsTnI demonstrated an AUC of 0.74 for predicting major cardiac events during ICU admission. The optimal Youden index corresponded to an hsTnI threshold of 12.9 ng/L. This cut-off yielded a sensitivity of 73% and specificity of 72% for detecting significant cardiac events.The positive predictive value was 9% and negative predictive value was 99%. The positive likelihood ratio was 2.61 and negative likelihood ratio was 0.37. A hsTnI concentration inferior or equal to the minimal detectable threshold of 5 ng/L excludes a cardiac complication with a sensitivity of 82%, resulting in a negative predictive value of 98% and a negative likelihood ratio of 0.32. Concerning 30-day mortality, initial hsTnI level had an AUC of 0.75. The optimal Youden index corresponded to the same threshold of 12.9 ng/L. This threshold yielded 74% sensitivity and 72% specificity for predicting 30-day mortality. The positive predictive value was 15% and negative predictive value was 98%.

#### Factors related to major cardiac events

For analysis, patients were stratified according to the occurrence of major cardiac events during ICU stay, as previously defined. Between-group comparisons are presented in Table 3. In univariate analysis, patients who developed major cardiac events had significantly higher SAPS II scores, were more likely to require vasopressor support, renal replacement therapy, and invasive mechanical ventilation. They also had longer ICU stays and were older. Although hsTnI concentrations tended to be higher in the cardiac event group, this difference did not reach statistical significance. Subsequently, a multivariate analysis was conducted to identify independent factors associated with the occurrence of major cardiac events during ICU admission. To assess the influence of hsTnI variation, a second measurement obtained during admission was analysed when available: patients were classified as having increasing hsTnI if the second value exceeded the first, or decreasing hsTnI if it was equal to or lower. Owing to the retrospective design, intervals between measurements were at the discretion of the treating physician and thus not standardised. Of the 1,749 patients, 661 had a second hsTnI value documented, with a median measurement interval of 8.9 hours (IQR 9.0 h). The results of the multivariate logistic regression are displayed in Table 4. In this multivariate analysis, neither the initial hsTnI concentration nor an increase in hsTnI on a subsequent measurement independently predicted cardiac complications during the ICU stay. Conversely, both higher SAPS II scores and advancing age at admission were significantly linked to an elevated risk of cardiac complications, with each additional SAPS II point and each year of increased age corresponding to an approximate 3% rise in risk.

**Table 3:** Univariate comparisons between patients with and without major cardiac events during ICU stay. Continuous variables are expressed as median (interquartile range) and categorical variables as n (%), with p values from Welch’s t test for continuous data and Pearson’s chi‑squared or Fisher’s exact tests as appropriate

Characteristic	No cardiac event	Cardiac event	p-value^*2*^
	N = 1,684^*1*^	N = 64^*1*^	
Initial hsTnI (ng/L)	5 (5, 19)	34 (10, 166)	0.064
Age (years)	42 (26, 58)	65 (49, 81)	<0.001***
Sex (Male)	1,281 (76%)	47 (73%)	0.6
ICU length of stay (days)	4 (1, 8)	9 (4, 18)	<0.001***
SAPS II Score	23 (15, 38)	48 (32, 67)	<0.001***
Vasopressor drug use	506 (30%)	39 (61%)	<0.001***
Renal replacement therapy	60 (3.6%)	10 (16%)	<0.001***
Invasive ventilation	429 (25%)	37 (58%)	<0.001***
30-day mortality	94 (5.6%)	17 (27%)	<0.001***

^*1*^Median (Q1, Q3); n (%); ^*2*^Welch Two Sample t-test; Pearson’s Chi-squared test; Fisher’s exact test

## Discussion

Our study evaluated the ability of high-sensitivity troponin I to predict major cardiac event in patients with chest trauma using continuous variables rather than predetermined cut-off values, in order to determine more appropriate standards in the setting of trauma care. This capacity could be categorised as moderate, with an AUROC of 0.74 to predict cardiac complication during ICU stay, and 0.75 to predict mortality at 1 month. The threshold of 12.9 ng/L provides the best Youden index, with a sensitivity of 73% and a specificity of 71%. This predictive capacity was inferior to that described in the literature for standard troponin I and T assays [10]and is probably the result of our more restrictive definition of cardiac complications. When several threshold values are explored, our analyses highlight the most demonstrated utility of its use which is the exclusion of a cardiac complication [11, 12] with a sensitivity 82% 3 when the troponin value is negative ($\le$ 5 ng/L), resulting in a negative predictive value of 98% and a negative likelihood ratio of 0.32 with the prevalence of cardiac events of 3.7%. In our analysis of risk factors for cardiac complications, univariate analysis revealed significant differences for indicators reflecting overall patient severity: SAPS II, catecholamine use, mechanical ventilation and dialysis. However, we observed that these results likely reflect a more critical physiological state in patients with cardiac complications. Therefore, we performed a multivariate analysis to assess the impact of hsTnI and its variations, excluding patient intrinsic severity factors. This analysis revealed no significant association between initial troponin level or its increase on a second measurement and the risk of cardiac complications during ICU stay. The study of the increasing hsTnI parameter was motivated by the common clinical practice consisting of performing troponin assays at frequent intervals to detect possible cardiac injuries in the case of hsTnI increase. Our results do not support this strategy. However, factors reflecting patient physiological state, such as age and SAPS II score appeared to have a significant influence on the risk of developing cardiac complications. The interpretation of all these results depends mainly on the assessment criteria we used. Because there is no consensus definition of BCI in the literature, its incidence is strongly dependent on the events included. Some studies included a wide variety of events, which may therefore not be specific to cardiac injury, such as episodes of arterial hypotension, or asymptomatic troponin elevation [13, 14]. Assessing the possibility of BCI during management of a trauma patient is essential, as the complications such as arrhythmia or structural abnormalities can be life-threatening. The EAST guidelines recommend screening for BCI by measuring troponin I and performing an ECG, with a recommendation for hospitalisation and continuous monitoring in case of abnormal results [5]. These recommendations are based on the measurement of troponin I using standard techniques, but the new "ultra-sensitive" techniques allow the detection of lower plasma thresholds, which may consequently increase the proportion of positive asymptomatic patients and modify the diagnostic strategy. In a 2019 study including a general emergency department population presenting without symptoms of myocardial ischaemia, 12.4% of patients had hsTnI values above the 99th percentile. A troponin elevation was strongly correlated with factors such as age, sex and renal function [8]. These technical developments required new studies to investigate the equivalence of these ultrasensitive assays in the clinical determination of BCI risk, which validated the use of hsTnI as a substitute for TnI [9], with the same diagnostic capacity, particularly in the rejection of the diagnosis of BCI in case of negative results using standard thresholds.

**Table 4:** Multivariable logistic regression identifying independent factors associated with major cardiac events during ICU admission. Covariates include log‑transformed initial hsTnI (ng/L), an indicator for increasing hsTnI on the second measurement, SAPS II score, and age, with odds ratios and 95% confidence intervals reported. All patients with at least 2 hsTnI measurements were analysed (n=661). “Increasing hsTnI” denotes a second value greater than the first

Characteristic	OR^*1*^	95% CI	p-value
hsTnI (log ng/mL)	1.16	0.97, 1.37	0.10
Increasing hsTnI	1.00	0.43, 2.55	>0.9
SAPS II Score	1.02**	1.01, 1.04	0.002
Age	1.02**	1.01, 1.04	0.003

^1^*p<0.05; **p<0.01; ***p<0.001 Abbreviations: CI = Confidence Interval, OR = Odds Ratio

In our opinion, a strength of our study was the strict definition of cardiac-event, which only includes complications that are judged clinically relevant in the critical care setting, and may induce a change in patient acute management. This definition excluded asymptomatic disorders that were detected incidentally. Complications were identified using the diagnostic coding system used in French hospitals (PMSI), which is based on the ICD-10 classification. The screening of these complications has been adjusted on the basis of medical discharge reports, where only clinically significant events are reported. Consequently, we found a cardiac complication incidence of 3.7%, which is lower than the rate observed in the literature [15–17]. Another major strength of our study is the inclusion of all patients hospitalised in our critical care departments for chest trauma without interruption over a period of 5 years. All patients meeting chest trauma criteria were included exhaustively, which provides a good reflection of epidemiology of cardiac complications in these patients. Moreover, the method used to measure ultrasensitive troponin I remained unchanged during all the study period, meaning that the values can be compared uniformly. However, our study suffers from several limitations, mainly due to its retrospective design. First the available data were limited, and we were unable to retrieve any information on the severity of trauma, such as "Injury Severity Score" (ISS). This score is used to standardise the evaluation of patients admitted for severe trauma and is significantly linked to mortality [18–20]. In addition, some studies have demonstrated that attempting to classify severity of trauma using ICD-10 diagnoses was not accurate [21]. We therefore chose to minimise this limitation by using another global severity score, the SAPS II score, which is a model for predicting mortality in intensive care [22, 23]. This score was chosen although it is not specific to trauma patients, as it reflects a physiological and metabolic severity. Secondly, no specific recommendations were issued regarding in-ICU management, particularly the intervals between troponin measurements or the use of additional investigations such as ECG or echocardiography, and these decisions were left to the discretion of the treating physician. This lack of standardisation complicated the interpretation of absolute or relative variations in hsTnI without extrapolation methods. To reduce this bias, we chose to interpret the variations in ultra-sensitive troponin using a binary variable based on analysis of the first two values. We believed that this method reflected well the common interpretation, which consists of detecting any increase in troponin as a warning signal of cardiac injury. Furthermore, because our study focused on the ICU stay, it did not capture delayed complications that may have remained asymptomatic during hospitalisation or late morbidity occurring after discharge. Likewise, as patients in this cohort were critically ill, our findings regarding the performance of hsTnI may be less generalisable to populations with a lower pre-test probability of cardiac complications. Moreover, the SAPS II severity scoring system is tailored to the ICU, and may not be readily applicable as a screening tool in settings where close monitoring is less feasible. Finally, no patient medical history could be collected due to the lack of such information in our database. Consequently, no analyses were performed on the effect of past medical history of cardiovascular disease on the risk of cardiac complications, although it is reasonable to suppose their potential contribution to such complications. Our study raised pertinent questions about the use of ultrasensitive troponin as a predictive biomarker in chest trauma patients. Ruling out cardiac injury when troponin level is undetectable on admission seems appropriate, as it could quickly lead to optimisation of care by reducing the amount of monitoring required. This initial determination is simple to integrate into the routine biological tests performed on admission of trauma patients. In contrast, clinical decisions based on positive ultrasensitive troponin results appeared more debatable and complex. Although our study did not include a medico-economic analysis, it is possible that consideration of such positive results could lead to additional costs in terms of financial and human resources, in particular unnecessary hospitalisations or prolonged stays in intensive care, or accumulation of additional investigations attempting to confirm an injury. The cognitive bias of systematically monitoring abnormal results until they normalise contribute to these extra costs. Based on our study results, it is legitimate to debate the rationale of this practice in chest trauma patients, since variations in hsTnI do not demonstrate any predictive utility. Future studies should include standardised troponin measurement intervals (e.g., at admission, 6 hours, and 24 hours), comprehensive cardiovascular history collection, and cost-effectiveness analyses to better define the clinical utility of hsTnI monitoring in chest trauma patients. Such an approach would allow a more precise evaluation of troponin variation. It would be then possible to identify clusters of patients defined by specific troponin variations, which would be linked to a high or low risk of cardiac complication.

## Conclusion

The study found that cardiac events are uncommon in critical care chest trauma patients, with an incidence of 3.7%. The initial value of hsTnI provides a moderate predictive performance with an AUROC of 0.74. Best Youden index corresponds to a threshold of 12.9 ng/L with a sensitivity of 73% a specificity of 71%. Increasing hsTnI on a second measurement did not appear to be predictive of cardiac complications, contrary to factors reflecting physiological state like age or SAPS II score. Further studies are needed to determine whether specific patterns of high sensitivity troponins variations are associated with cardiac events.

## Data Availability

No datasets were generated or analysed during the current study.
